# Regional health diplomacy as a health promotion strategy for robust health systems

**DOI:** 10.34172/hpp.025.44898

**Published:** 2025-07-15

**Authors:** Vijay Kumar Chattu, Hamid Allahverdipour

**Affiliations:** ^1^United Nations University Institute on Comparative Regional Integration Studies (UNU-CRIS), Potterierei 72, 8000 Bruges, Belgium; ^2^Department of Epidemiology and Biostatistics, Semey Medical University, Semey 071400, Kazakhstan; ^3^Department of Community Medicine, Faculty of Medicine, Datta Meghe Institute of Medical Sciences (DMIMS), Wardha, 442107 India; ^4^Department of Health Education & Promotion, Tabriz University of Medical Sciences, Tabriz 14711, Iran

 The World Health Organization (WHO) at the Ottawa Charter for Health Promotion in 1986 during the first International Conference on Health Promotion defined health promotion as “the process of enabling people to increase control over their health and its determinants, and thereby improve their health”.^1^ Prior to that in 1984, the WHO Regional Office for Europe defined health promotion as “the process of enabling people to increase control over, and to improve, their health”.^2^ The Alma-Ata Declaration and Primary Healthcare for All in 1978 acknowledged and stressed the need for immediate action by all governments, non-state entities, and many social and economic players outside of the health sector in achieving an acceptable level of ‘health for all people’.^3^

 The Centers for Disease Control and Prevention has cited health diplomacy as a way to improve trust and provide resources for health protection and promotion globally.^4^ According to Blumenthal and Schlissel, “Health diplomacy is a means of self-preservation in an increasingly interconnected global community. It also offers a much-needed opportunity for building bridges between the governments of the world and the private sector, synergizing efforts of nongovernmental organizations (NGOs) and allowing them to work together to improve public health.”^5^ Health diplomacy therefore integrates health concerns into foreign policy and leverages diplomatic resources to advance global health goals. The contemporary global health environment draws on a 160-year legacy, with the goal of establishing systems to promote health and combat disease across national borders.^6^

 Applying this broader view of health promotion, Chattu has highlighted that the people who work in the field of health diplomacy/regional health diplomacy (RHD)/global health diplomacy address the national, regional and global health challenges such as infectious diseases, noncommunicable diseases (NCDs) and other health threats by promoting health as a foreign policy priority with an ultimate goal of attaining the health and well-being of all its citizens. The research further concluded that RHD can be used as a tool for promoting peace, health and influence the change of unhealthy behaviours in the prevention of NCDs through enacting policies that are developed through active participation of all the stakeholders in the Caribbean region.^7^ Given this context, this editorial provides a concise, crisp overview of RHD and how it can play a strategic role in health promotion thereby contributing to the regional health security and regional socioeconomic development.

## Link between health promotion and health diplomacy

 However, according to the Ottawa Charter for Health Promotion, health promotion highlights four core components: i) “is not just the responsibility of the health sector but goes beyond healthy lifestyles to well-being”; ii) “focuses on achieving equity in health”, iii) “aims at making [political, economic, social, cultural, environmental, behavioral and biological factors] favorable through advocacy for health” and iv) “demands coordinated action by all concerned: by government, by health and social organizations”.^1^ Sir Michael Marmot, who championed the significance of tackling the socioeconomic determinants of health, disclosed that in the previous ten years, health disparities have not been a priority, and there has not been a national plan in place.^8^ One of the main reasons could be due to the lack of political commitment and financing among the various things. Therefore, to garner the political commitment, it is essential to frame the issues at national and regional level requiring the need for health diplomacy/RHD.

 In this globalized world, health becomes increasingly important in trade agreements, development strategies, security policies, and foreign policy. Therefore, as highlighted by the WHO, health is increasingly acknowledged as a fundamental objective of foreign policy and a significant factor in development, peace, poverty alleviation, social justice, and human rights.^9^ Given how linked our world is and how quickly infectious diseases can spread across continents, global health is considered as a shared global concern. Media-spread lifestyle elements can quickly influence the global population, and knowledge can be transferred instantly.^6^ Globalization of health resulted in a shift in disease patterns, improved understanding of the social and economic determinants of health and increased diversity of institutional actors. For example, many social and economic determinants that are outside the purview of the health system drive the risk factors for NCDs that have a greater impact on people, households, and communities. These determinants include gender inequality, employment conditions, globalization, trade, education, urbanization, and poverty among others.^7^

## Regional health diplomacy: Why, what and how?

 Regional challenges such as disease outbreaks/ epidemics, rising burden of NCDs, war, displacement, climate migration, instability and insecurity due to geopolitical issues.^10^ There is a great need for countries to strengthen RHD because many of the development difficulties they encounter are directly related to health, and because man-made and humanitarian catastrophes affect them disproportionately. For example, in addressing NCDs epidemic, an effective and sustainable strategy requires cooperation and collaboration among national governments, the private sector, and civil society at international, national, and local levels. It was stressed that global solidarity, multilateralism, international cooperation, effective regional governance mechanisms for global health required for NCDs prevention could all happen through successful RHD.^7^

 RHD encompasses a number of stakeholders, ministerial departments and disciplines, including public health, international law, international affairs, economics, Information Technology, and management, with the goal of shaping the regional health policy environment and promoting peace through successful negotiations.^7^ RHD focuses on using health-related issues to foster cooperation and improve health outcomes within a specific geographic area. It involves various actors, including governments, international organizations, and civil society, working together to address shared health challenges and promote regional stability. This approach recognizes that health is not solely a national issue and that regional cooperation can lead to more effective and sustainable solutions.^11^ RHD can serve to establish political and community will, coordinate responses, enhance access to children, and create the conditions for community and political engagement.^10^

## Framing health agendas at the regional platforms

**Figure 1:**
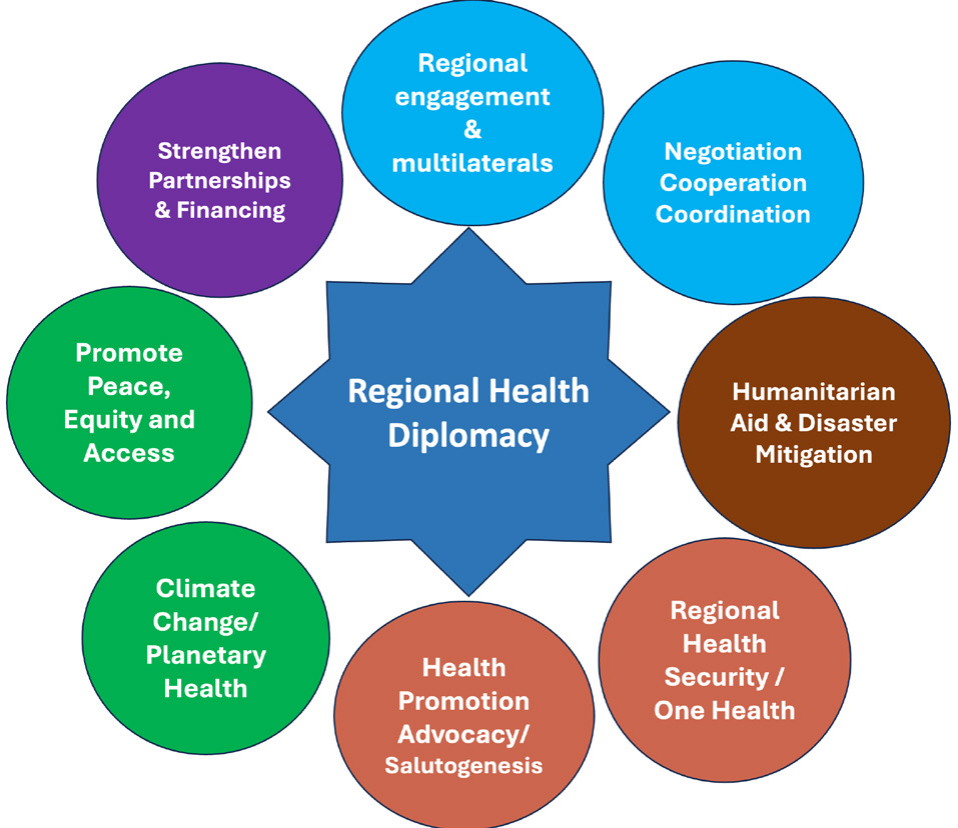
Various useful activities and themes that can be addressed through Regional Health Diplomacy

 The topic of health has become a priority issue that is being explored by numerous players outside of the WHO in order to form global policy for health determinants. Thus, health has been a focus of UN Summit diplomacy encompassing the G8, G20, BRICS, and the EU.^12^ Foreign ministries are becoming increasingly active in the health domain, which is used for soft power, creating security policies, and negotiating trade agreements because it touches on concerns of nation development and economic interests. The new public-private partnerships, coalitions, and various forms of regional cooperation among low-middle-income countries (LMICs) have put into question the need of RHD.^7^ RHD plays a significant role in promoting sustainable development by framing health as a critical social and economic issue and addressing health challenges both individually and collectively as a region. It can assist nations in safeguarding shared interests and in formulating positions on issues of mutual concern, including health security,^13^ health promotion, disease control, access to medicines and technologies,^14^ food security, water, and the post-2015 agenda.^9^ Besides, RHD can be a tool to strengthen the regional partnerships, cooperation, engagements and commitment for the overall development of the regions which are also linked/ aligned with the goals of various UN sustainable development goals (SDGs).^15^ A summary of these activities and purposes are shown below (Figure 1).

 Therefore, RHD becomes very critical in framing health in the regional health agendas. Health promotion involves not just individual change of behavior but also modifying the social and environmental determinants of health. Besides, through RHD, nations/ states can further push for promoting successful strategies such as One Health^16^ or “Health in All Policies” (HiAP) which refers to actions that incorporate health into all public policies should be on the main agenda of all nations for bridging health inequities.^17^ This statement made at Adelaide engages leaders and policy makers at all levels - local, regional, national, and international. It highlights that government objectives are best attained when health and well-being are prioritized in policy making across all sectors. Therefore, the regions can address issues that require global action and cross-border collaboration such as humanitarian health aid, NCDs, Climate Change and growing antimicrobial resistance (AMR). Through regional cooperation, and governance and successful negotiations, RHD can bring in new creative financing structures collectively to address the regional requirements with the cooperation of regional institutions, multilaterals, philanthropies, INGOs and their pooled resources/commitments.

## Key variables and challenges of regional health diplomacy

 There are many internal and external factors that can impact the outcomes of RHD as the stakeholders and players such as public, private, international actors and civil society form a set of few variables. However, in this interconnected globalized world, the domestic indicators such as the development status, country development index, gross domestic product, equity, accessibility issues, governance structures as well as national interests play a significant role. Some of the variables of RHD at national and regional levels are listed below (Table 1).

**Table 1:** List of some critical variables of regional health diplomacy at national and regional levels

**Dimension**	**National**	**Regional**
*Priorities*	National interests; level of political commitment	Regional aspirations and priorities; level of political commitment
*Governance*	Type of government and governance	Governance structures;
*Power *	Hierarchy and Authority	Power dynamics through influence of hard power. E.g. Military, Economy, Technology, etc.
*Health*	Health priorities; Technical capacities	Public health emergencies or regional health policy frameworks
*Equity and Access*	Access to health care and other public policies to ensure equity and equality	International/ bilateral agreements, frameworks for cooperation for sharing resources and to address gaps
*Financing*	Domestic budgets	Investment strategies
*Legal frameworks*	Law and Regulations	International norms; regional frameworks
*Partnerships*	Civil society engagement and NGOs	Civil Society Organizations, International NGOs and Stakeholder alliances

Source: Prepared by the authors (adapted from Chattu VK, 2025)^7^

 The role of health diplomats/health experts in RHD is very critical to design and negotiate regional frameworks and agreements through their help and active engagement with traditional diplomats in a collaborative and coordinated way to address global health challenges. Some notable examples include- Port of Spain declaration^18^ and Bridgetown declaration in the CARICOM,^19^ establishment of a continental African Medicines Agency,^20^ ASEAN’s commitment for NCDs, emerging threats, strengthening health systems and food safety.^21^ From the Central Asian region, the Central Asia Commitment to One Health Framework for Action was held at Almaty in Kazakhstan to address three high-level goals in the region: i) pandemic prevention and preparedness, ii) resilience of food systems, and lastly iii) improving regional trade and the competitiveness of agriculture.^22^

## Conclusion

 Health promotion involves public policy which addresses social/health determinants such as family income, housing, food security, employment status and quality of working conditions which can be framed and addressed through RHD. Since health promotion involves not just individual change of behavior but also modifying the social and environmental determinants of health, there is a great need for promoting multidisciplinary RHD which also strengthens the bonds between nations and contribute for regional development and effective governance to achieve the global goals. Promoting effective policies such as “Health in All Policies” should be on the main agenda of all nations for bridging health inequities and strengthening health systems. RHD as a health promotion strategy is critical to strengthen the interaction and coordination across health, foreign policy, and other areas at the regional level. Enhancing health, akin to education, addressing equity gaps are essential for national security and regional socioeconomic development which should be the top priority in this multipolar world with weakening multilateral world amid the current geopolitical scenario with proliferation of more nationalistic agendas.

## Competing Interests

 Vijay Kumar Chattu is a member of the editorial board, and Hamid Allahverdipour is the Editor-in-Chief of *Health Promotion Perspectives*.

## Ethical Approval

 Not applicable.
